# Heat-stress priming and alternative splicing-linked memory

**DOI:** 10.1093/jxb/ery111

**Published:** 2018-04-26

**Authors:** Ravi P Sanyal, Hari S Misra, Ajay Saini

**Affiliations:** 1 Molecular Biology Division, Bhabha Atomic Research Centre, Trombay, Mumbai, Maharashtra, India; 2 Homi Bhabha National Institute, Anushaktinagar, Trombay, Mumbai, Maharashtra, India

**Keywords:** Acquired tolerance, alternative splicing, heat-stress memory, heat-stress priming, intron retention, stress response

## Abstract

This article comments on:

**Ling Y, Serrano N, Gao G, Atia M, Mokhtar M, Woo YH, Bazin J, Veluchamy A, Benhamed M, Crespi M, Gehring C, Reddy ASN, Mahfouz MM.** 2018. Thermopriming triggers splicing memory in Arabidopsis. Journal of Experimental Botany **69,** 2659–2675.

**Stress-induced priming and associated memory is an intriguing adaptive response in plants, and one with important implications for crop development. Ling *et al.* (2018) carried out a comprehensive RNA-Seq analysis of gene expression and splicing events in heat-stress primed and non-primed plants, revealing alternative splicing as a novel and vital component of heat-stress priming induced memory. The splicing-linked memory programmed during the priming phase is important for ensuring the availability of correctly spliced transcripts/proteins critical for enhanced tolerance.**

Heat stress is one of the most important abiotic threats affecting agricultural productivity worldwide, with severe impacts on major crop yields (Bray *et al.*, 2000; Zhao *et al.*, 2017). Research facilitating the development of stress-tolerant crops is therefore vital, and utilizes different approaches to enable plants to adapt, survive and perform to their full potential under stress conditions. These include conventional/marker-assisted breeding and/or advanced transgenic methods involving genome editing (Savvides *et al.*, 2016). While breeding methods are time-consuming and require a stress-tolerant source (in the same or a closely related species), a transgenic approach enables introgression of a desired ‘monogenic’ trait much more quickly. However, this needs perfectly optimized protocols for the specific crop and is difficult for multigenic traits (such as abiotic stress tolerance), and moreover genetically modified (GM) crops are associated with lower acceptability by society.

Adaptation strategies suitable for different crops and regions offer a simple alternative for the development of crops tolerant to abiotic stress, ensuring food security (Zhao *et al.*, 2017). ‘Priming’, initially used in the context of pathogen defence (Conrath *et al.*, 2002), allows such acquired stress tolerance and offers a number of advantages: there is no introgression of an external genomic entity and it involves sub-lethal stress-mediated reprogramming of the molecular machinery to achieve enhanced tolerance (Lämke and Bäurle, 2017); it is relatively fast; it is applicable for diverse stress conditions; and, with some optimization, it is capable of enhancing tolerance in a range of crops.

## Stress-priming induced memory involves complex molecular mechanisms

Exposure to sub-lethal heat stress enables plants to acquire thermotolerance (Box 1), a relatively well-conserved mechanism among different organisms (Mittler *et al.*, 2012). The process involves an initial priming phase and a distinct heat-stress memory state that remains active for several days (Charng *et al.*, 2006; Charng *et al.*, 2007; Lämke and Bäurle, 2017). Although a number of molecular mechanisms of heat-stress priming are well established for plants, the true nature of heat-stress memory is less clear (Bäurle 2016). Nevertheless, we do know that heat-stress memory genes show sustained induction, ensuring high expression levels of relevant transcripts, and enhance the stability and/or activity of important proteins (Charng *et al.*, 2006, 2007; Nishizawa *et al.*, 2006; Meiri and Breiman, 2009).

**Box 1. Priming-based enhanced heat-stress tolerance and modes of alternative splicing**

(a) Heat-stress (HS) priming involves exposure to sub-lethal heat stress to induce complex reprogramming of cellular mechanisms to achieve enhanced stress tolerance. The overall mechanism can be divided into (i) stress priming, (ii) memory establishment, and (iii) adaptive responses. Heat-stress priming mediated memory establishment is crucial for survival of plants in a second episode of lethal heat stress.
(b) Intron-containing eukaryotic genes undergo alternative splicing (AS) in multiple ways. These include exon skipping (ES), 5´ alternative splice site recognition (5´ASS), 3´ alternative splice site recognition (3´ASS), intron retention (IR) and mutually exclusive exons (MEE). Alternative splicing is always operative, and produces several RNA/protein isoforms with functional significance in a diverse array of cellular functions, many of which are not completely understood. It is affected by developmental and environmental cues, and is important for cellular responses in such conditions. CS, constitutive splicing; E, exon; I, intron. Different alternative splicing events are indicated by differently coloured arrows.

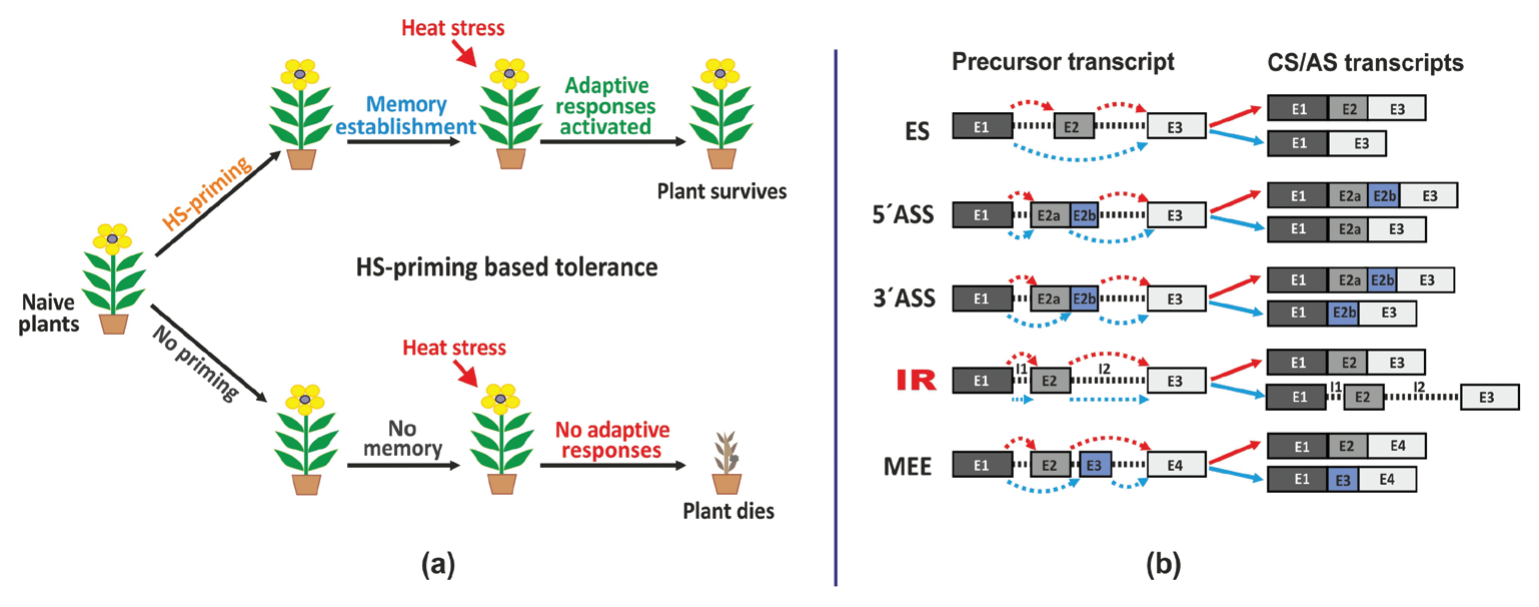

The initial phase of the heat-stress response (including memory) involves a complex interplay between several transcription factors, including heat-stress transcription factors (HSFs), controlling the expression of heat-stress response genes (Ohama *et al.*, 2017), and specific chromatin/histone modifications (e.g. H3 lysine methylation) that mark memory genes for rapid re-induction during subsequent heat stress (Lämke *et al.*, 2016; Ohama *et al.*, 2017) as well as other stress conditions (Sani *et al.*, 2013). At the protein level, establishment of heat-stress memory involves several proteins, including heat shock proteins (HSPs) with chaperone activity (Wu *et al.*, 2013). Stress-primed memory also involves small RNAs that regulate genes involved in reprogramming growth, development and differentiation under stress (Sunkar, 2010; Stief *et al.*, 2014). In this already complex scenario, Ling *et al.* (2018) have now shown the involvement of alternative splicing as a novel and integral component in establishing heat-stress priming induced memory in Arabidopsis.

## Splicing in eukaryotes: a highly versatile, multifaceted mechanism

Splicing, an intriguing post-transcriptional mechanism, generates mature RNA transcripts from intron-containing eukaryotic genes in multiple ways (Box 1). It has evolved from simple self-splicing introns (reminiscent of an early RNA world) to a highly complex ribonucleoprotein (RNP) machine, the spliceosome. Most constitutively spliced genes are also alternatively spliced in response to developmental/environmental cues to enhance transcriptome and proteome diversity for different functions (Reddy *et al.*, 2012). In recent years, the availability of high-throughput RNA-Seq datasets coupled with refined bioinformatics predictions have enhanced our understanding about its prevalence and importance in animals and plants (Marquez *et al.*, 2012; Reddy *et al.*, 2012).

Alternative splicing events such as intron retention (IR), exon skipping (ES), alternative splice site recognition (5′ASS and 3′ASS) and mutually exclusive exons (MEE) generate RNA/protein isoforms with altered stability, activity and cellular localization (Reddy *et al.*, 2013). Alternative splicing events are not equally prevalent among organisms; IR events are most predominant in plants, contributing to ~40% of their total (Syed *et al.*, 2012). IR isoforms are generally subject to nonsense-mediated decay (NMD), a cytosolic decay pathway (de Lima Morais and Harrison, 2010), but in plants they often escape this fate suggesting a different mode of regulation and function (Kalyna *et al.*, 2012). Such new functional modes of IR transcripts have been reported in gametophyte development (Boothby *et al.*, 2013) and neurogenesis (Yap *et al.*, 2012), and these reports also suggest the importance of IR-type alternative splicing events in development and stress responses (Reddy *et al.*, 2013). The study of Ling *et al.* (2018) goes on to show that heat-stress priming enables plants to ‘remember’ constitutive splicing patterns right after relief from second or recurring exposure to heat stress and generate correct transcripts/proteins that ensure survival.

## Heat-stress priming and priming-induced memory: is splicing a vital connecting link?

Ling *et al.* (2018) carried out an extensive comparative analysis of the transcriptomes of heat-stress primed and naive plants. Extensive analysis of splice junctions identified 30% novel splice junctions. Simultaneous analysis of alternative splicing events and gene expression revealed an overall higher prevalence of IR events, also in certain categories of differentially expressed genes. The most remarkable and novel finding was the way splicing-linked memory establishment was mediated by enhanced IR events during the heat-stress priming phase, and this newly identified memory function ensured availability of correctly spliced transcripts (and proteins) needed for survival of plants during subsequent heat stress (Box 2).

**Box 2. Stages of the heat-stress response and IR-type alternative splicing events**

Exposure to sub-lethal heat stress results in priming of naive plants, which establishes splicing-linked heat-stress memory. Splicing repression and enhanced intron retention (IR) events result in a greater abundance of IR-precursor RNA isoforms. Subsequent exposure to heat stress activates splicing-linked memory, which executes correct splicing and ensures the availability of stress-responsive RNA/proteins resulting in an effective adaptive response that ensures survival of plants. AS, alternative splicing; CS, constitutive splicing; E, exon; I, intron. Different alternative splicing events are indicated by differently coloured arrows. Arrow thickness indicates abundance.

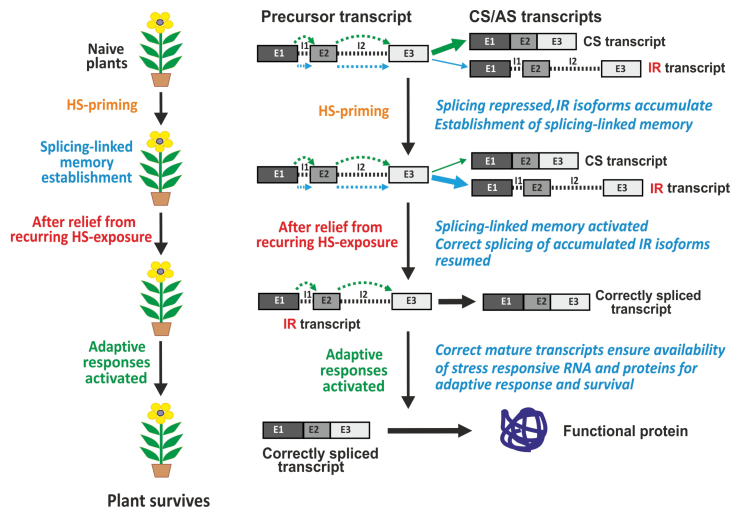

Abiotic stress tolerance involves the complex interplay of a number of factors which also include higher basal levels (Taji *et al.*, 2004) and rapid up-regulation (Kawasaki *et al.*, 2001) of stress-responsive genes/proteins for an effective adaptive response and survival. Similarly, the IR-mediated accumulation of unprocessed transcripts (during heat-stress priming) followed by correct processing by splicing-linked memory ensure their timely availability for an appropriate stress-adaptive response. Moreover, this phenomenon has the capability to modulate expression of many genes in a uniform manner.

## Perspectives: basic and applied research

Alternative splicing signifies that multiple coding genomes can exist within a single genome, and these events are dynamically modulated in response to different cues, offering immense possibilities for the functioning of the cellular machinery. However, we only know the significance of 60–70% of the coding part of the basic genome, and much (beyond sequence similarity) about the alternative components is unknown.

The work of Ling *et al.* (2018) throws up new questions. Are all introns retained (as in a completely unprocessed isoform) or do some have a higher chance of retention? If some are preferentially retained, are certain splice junction(s) or intron sequence/secondary elements involved? Such associations (if found) may be useful for engineering the intron regions of desired gene(s) for expression during the memory establishment phase. Furthermore, it will be interesting to investigate whether the predominance of IR-type alternative splicing events in plants (compared with animals) has evolved as a stress-tolerance mechanism due to their sessile nature and continuous exposure to stress.

The extent of alternative splicing events other than IR (e.g. 5´ASS, 3´ASS) in establishing stress memory is not well known. However, many such isoforms with altered/missing interacting properties may contribute indirectly. SR proteins involved in splice junction recognition undergo alternative splicing to generate isoforms (Reddy, 2004); these may serve as dominant-negative regulators (Staudt and Wenkel, 2011), or recognize correct junctions (or conceal incorrect junctions) for splicing function under stress and be a component of splicing-linked memory. Equally important will be understanding the exact mechanism whereby splicing machinery is shuttled between repressed (during priming phase) and activated (during heat-stress phase) states.

Another aspect of the alternative splicing linked heat-stress memory phenomenon is how the correct splicing pattern is remembered, enabling the generation of mature molecules during the stress phase. Does it involve sequence/secondary elements of retained intron regions or factors that make intron regions of precursors accessible for correct processing?

It is also important to determine the fate of negative regulators of splicing during the heat-stress priming phase. As the abiotic stress response is an interplay of positive and negative regulators it will be interesting to see how IR events affect the levels of negative regulator precursors. Is splicing-linked memory in other stresses divergent or convergent?

The simplicity of priming and associated memory can be utilized to enhance stress tolerance of various crops, but the effect of heat-stress ramp rate (rate of increase in temperature per unit time) on priming, and duration for memory, also need to be investigated and optimized for different crops. Certain isoforms can be identified as an ‘indicator panel’ to confirm memory establishment and simplify it for in-field applications. It may also be useful for optimizing parameters for different plants/stresses and for identification of chemical elicitors/agents for establishing memory. Several chemical agents are known to induce stress-priming of crops (Savvides *et al.*, 2016), and these should be re-evaluated to see if any act via alternative splicing-linked memory as observed for heat-stress priming (Ling *et al.* 2018). Such agents would be immensely useful for stress-memory establishment in the field.
